# Pomalidomide-induced acute eosinophilic pneumonia: a case report

**DOI:** 10.1186/s12890-026-04438-1

**Published:** 2026-06-20

**Authors:** Mathieu Megevet, Jessica Freitas Pereira, Jeanne Vervier, Pierre-Yves Lovey, Vincent Soubeyran, Pierre-Olivier Bridevaux

**Affiliations:** 1 Service de pneumologie, Centre Hospitalier du Valais Romand, Valais, Switzerland; 2 Service de pneumologie, Hôpitaux Universitaires de Genève, Genève, Switzerland; 3 Université de Genève, Genève, Switzerland; 4 Service de médecine interne, Centre Hospitalier du Valais Romand, Valais, Switzerland; 5 Service d’hématologie, Centre Hospitalier du Valais Romand, Valais, Switzerland; 6 Service de radiologie, Centre Hospitalier du Valais Romand, Valais, Switzerland; 7 CHVR, Avenue du Grand-Champsec 80, Sion, 1950 Switzerland

**Keywords:** Pomalidomide, Drug-induced lung injury, Acute eosinophilic pneumonia, Pulmonary toxicity, Multiple myeloma

## Abstract

Eosinophilic pneumonia secondary to drug exposure is a recognized but infrequent complication associated with a variety of medications, including oncological therapies. Among immunomodulatory drugs, pulmonary toxicity has been described for thalidomide and lenalidomide but remains a rare complication.

Pomalidomide is a third-generation immunomodulatory agent used in relapsed or refractory multiple myeloma. It has been authorized in Europe since 2013. To date, only a limited number of cases of pomalidomide-induced lung injury, and even fewer cases of eosinophilic pneumonia, have been documented in the literature.

We present a case of eosinophilic pneumonia attributed to pomalidomide in a 67-year-old patient receiving daratumumab, pomalidomide, and dexamethasone for relapsed multiple myeloma. The diagnosis was supported by compatible respiratory symptoms and imaging, bronchoalveolar lavage eosinophilia, exclusion of alternative causes, lack of clinical improvement with empirical antibiotics, and rapid improvement after pomalidomide withdrawal and systemic corticosteroid therapy.

Prompt recognition of this rare adverse event, discontinuation of pomalidomide, and corticosteroid therapy resulted in rapid clinical and radiological improvement, with sustained pulmonary function recovery at 12-month follow-up.

## Case presentation

A 67-year-old male with a history of relapsed multiple myeloma, previously treated with two autologous hematopoietic stem cell transplantations 8 and 5 years before, was receiving daratumumab, pomalidomide, and dexamethasone (DPD). He was referred to the emergency department by his general practitioner after one week of worsening dyspnea, non-productive cough, sweating, and chills.

Pomalidomide had been administered at a dose of 4 mg once daily on days 1–14 of a 21-day cycle. First respiratory symptoms had begun three months earlier, four weeks after DPD initiation, corresponding to a cumulative pomalidomide exposure of approximately 88 mg.

On admission, vital signs were notable for a blood pressure of 107/65 mmHg, heart rate of 65 beats per minute, respiratory rate of 15 breaths per minute, temperature of 36.5 °C, and oxygen saturation of 93% while receiving 4 L/min supplemental oxygen via nasal cannula. Laboratory investigations revealed a raised C-reactive protein level at 155 mg/L and peripheral eosinophilia with an absolute eosinophil count of 0.8 G/L. Historical eosinophil counts had been within the normal range.

A chest radiograph showed ill-defined bibasilar infiltrates, predominantly affecting the left lower lobe, with preservation of the costophrenic angles. Microbiological tests, including a respiratory virus polymerase chain reaction (PCR) panel, urinary antigen tests for Streptococcus pneumoniae and Legionella pneumophila, and blood cultures, were negative.

The patient was admitted to the internal medicine department and empirical antibiotic therapy with co-amoxicillin and clarithromycin was initiated. Clarithromycin was discontinued after 48 h in the absence of initial evidence for atypical bacterial infection.

**Fig. 1:**
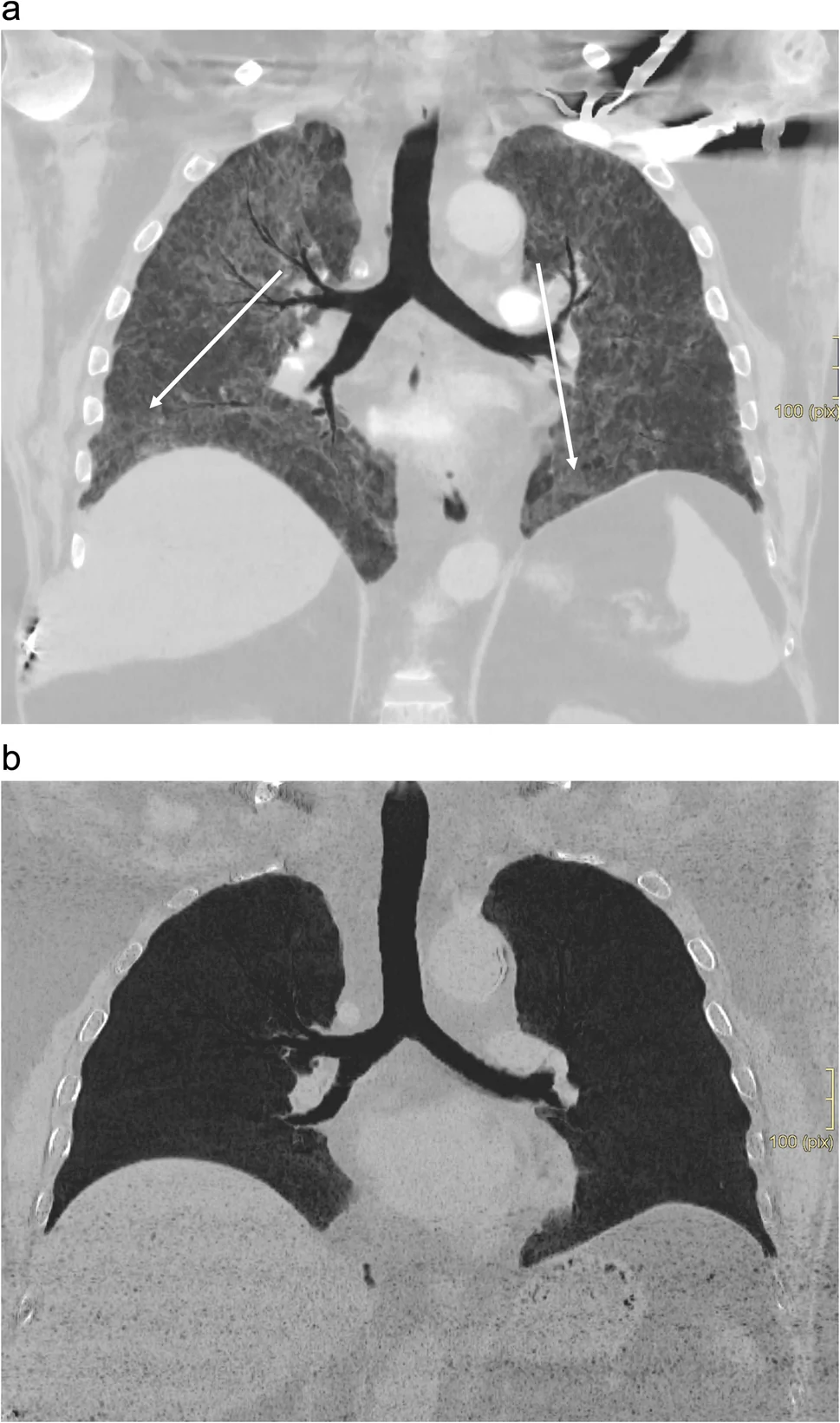
Chest CT at diagnosis and after treatment **a** Coronal CT image at diagnosis showing bilateral ground-glass opacities and intralobular reticulations, highlighted by arrows **b** One-month follow-up CT showing near-complete resolution after pomalidomide withdrawal and systemic corticosteroid therapy

Despite five days of antibiotic therapy, no clinical improvement was observed. A computed tomography (CT) scan of the chest (Fig. 1a) demonstrated diffuse bilateral intralobular reticulations with areas of ground-glass opacities. An arterial blood gas analysis revealed hypoxaemia without hypercapnia or acidosis.

Bronchoscopy with bronchoalveolar lavage (BAL) was performed, revealing a total cell count of 805,000 cells/mL, with a differential showing 32% eosinophils, 48% lymphocytes, 14% macrophages, and 5% neutrophils. Autoimmune testing, including anti-nuclear antibodies and anti-neutrophil cytoplasmic antibodies, was negative. Microbiological analysis of the BAL fluid was positive for Mycoplasma pneumoniae by PCR. PCR tests for respiratory viruses and Pneumocystis jirovecii were negative.

In light of the clinical presentation, imaging findings, BAL eosinophilia, and exclusion of other likely causes, a diagnosis of pomalidomide-induced eosinophilic pneumonia was suspected. By the time of discontinuation, the patient had completed four full cycles and had received an additional seven days of cycle 5, corresponding to a cumulative pomalidomide dose of 252 mg. Pomalidomide was permanently discontinued and no re-challenge was performed. The patient was started on systemic corticosteroid therapy with prednisone at 0.75 mg/kg daily (60 mg), which was subsequently tapered over eight weeks. Rapid clinical and symptomatic improvement followed corticosteroid initiation.

Pulmonary function tests performed at diagnosis (Table 1) showed lower values than the patient’s pre-illness baseline pulmonary function tests obtained one year earlier, with decreases of 830 mL in forced expiratory volume in one second (FEV1), 1.1 L in forced vital capacity (FVC), and 720 mL in total lung capacity (TLC).

At one-month follow-up, the patient reported no respiratory symptoms. Blood eosinophilia had resolved and repeat CT (Fig. 1b) showed near-complete resolution of prior abnormalities. Pulmonary function testing showed lung volumes remaining within the normal range, with a trend toward higher values and preserved gas transfer. Although gas transfer remained within the normal range, DLCO remained below baseline, while KCO was lower by percent predicted and Z-score, possibly reflecting residual alveolar-capillary dysfunction despite near-complete radiological resolution.

At 12-month follow-up, after completion of corticosteroid therapy, the patient remained free of respiratory symptoms, with no recurrence of pulmonary manifestations. Pulmonary function testing showed sustained improvement (Table 1).

**Table 1:** Evolution of pulmonary function tests at baseline, diagnosis, one-month follow-up, and 12-month follow-up

Parameter	Metric	Baseline	Diagnosis	1-month follow-up	12-month follow-up
FVC (L)	Value	5.52	4.42	4.52	4.86
	% predicted	124	101	105	114
	Z-score	1.60	0.08	0.33	0.89
FEV1 (L)	Value	4.55	3.72	3.71	3.95
	% predicted	134	112	113	122
	Z-score	2.30	0.77	0.85	1.39
FEV1/FVC (%)	Value	82	84	82	81
	% predicted	108	110	107	106
	Z-score	0.82	1.12	0.79	0.67
TLC (L)	Value	7.30	6.58	6.95	7.70
	% predicted	101	92	98	109
	Z-score	0.11	-0.67	-0.16	0.68
VC (L)	Value	5.47	4.34	4.59	5.20
	% predicted	113	91	98	112
	Z-score	1.01	-0.70	-0.17	0.95
DLCO (mmol/min/kPa)	Value	8.6	7.0	6.7	8.4
	% predicted	96	79	77	96
	Z-score	-0.23	-1.29	-1.43	-0.22
KCO (mmol/min/kPa/L)	Value	1.1	1.1	1.1	1.2
	% predicted	78	81	77	85
	Z-score	-1.40	-1.22	-1.49	-0.94

Predicted values and Z-scores were calculated according to Global Lung Function Initiative (GLI) reference equations

Abbreviations: *FVC* Forced vital capacity, *FEV1* Forced expiratory volume in one second, *TLC* Total lung capacity, *VC* Vital capacity, *DLCO* Diffusing capacity of the lungs for carbon monoxide, *KCO* Carbon monoxide transfer coefficient

## Discussion

Pomalidomide is a third-generation immunomodulatory agent used for the treatment of relapsed or refractory multiple myeloma and has also demonstrated activity in symptomatic Kaposi sarcoma [1–3]. Drug-induced interstitial lung disease has heterogeneous mechanisms and clinical patterns, including direct epithelial injury, immune-mediated inflammation, hypersensitivity-like reactions, and eosinophilic pneumonia [4]. Among immunomodulatory drugs, pulmonary toxicity has been reported with thalidomide [5, 6] and lenalidomide [7–9], with reported patterns including hypersensitivity pneumonitis, organizing pneumonia, interstitial pneumonitis, and eosinophilic pneumonia. The precise mechanisms of immunomodulatory drug-induced pulmonary toxicity remain incompletely understood, but BAL lymphocytosis or eosinophilia, steroid responsiveness, and improvement after drug discontinuation support an immune-mediated or hypersensitivity-like process in many cases.

Drug-induced eosinophilic pneumonia is usually supported by compatible symptoms and imaging, BAL eosinophilia, exclusion of alternative causes, and improvement after withdrawal of the suspected drug, with or without corticosteroid therapy [10, 11]. In our patient, accepted diagnostic elements were fulfilled: acute worsening of respiratory symptoms on a subacute background, new pulmonary infiltrates with ground-glass opacities and intralobular reticulations on CT, BAL eosinophilia exceeding 25% (32%), exclusion of likely infectious and autoimmune alternative causes, and clinical and radiological improvement after pomalidomide withdrawal and corticosteroid therapy.

In comparison with thalidomide and lenalidomide, pulmonary toxicity associated with pomalidomide appears less frequently reported. Published cases include interstitial pneumonitis, diffuse alveolar hemorrhage, lymphocytic or eosinophilic BAL patterns, and broader pulmonary toxicity [12–18]. Symptoms are usually non-specific, including dyspnea, cough, fever, hypoxaemia, and bilateral ground-glass or interstitial changes on CT, thereby frequently mimicking infection. Most reported patients improved after pomalidomide withdrawal, with or without systemic corticosteroids. Our case shares several features with these reports, including delayed respiratory symptom onset after pomalidomide initiation, bilateral CT abnormalities, BAL eosinophilia, lack of clinical improvement under empirical antibiotics, and rapid improvement after pomalidomide withdrawal and corticosteroid therapy.

The positive BAL PCR for Mycoplasma pneumoniae represents the main diagnostic confounder. The exact method was PCR performed on BAL fluid. A nasopharyngeal PCR performed five days earlier was negative. Serological confirmation was not performed, because the overall clinicobiological pattern was not considered consistent with active Mycoplasma pneumoniae as the primary driver, and because serology would not have reliably resolved causality in this immunocompromised patient after PCR detection. The isolated BAL PCR result could therefore represent clinically insignificant detection, contamination, or detection unrelated to the dominant pulmonary syndrome. More generally, nucleic acid detection of Mycoplasma pneumoniae in respiratory samples does not by itself establish symptomatic infection or causality [19].

Several arguments support exclusion of Mycoplasma pneumoniae as the primary cause of the pulmonary syndrome: the symptoms developed after pomalidomide initiation and progressed over several weeks; BAL showed substantial eosinophilia rather than a typical bacterial profile; autoimmune testing was negative; no other infectious cause was identified; the patient did not improve clinically during empirical antibiotic therapy; and improvement was rapid after pomalidomide withdrawal and systemic corticosteroid initiation. Although the short exposure to clarithromycin means that antibiotic non-response alone cannot exclude Mycoplasma infection, the full clinical, biological, radiological, and therapeutic pattern strongly supports pomalidomide-induced eosinophilic pneumonia as the most likely diagnosis.

A retrospective causality assessment using the Naranjo Adverse Drug Reaction Probability Scale yielded a score of 7, supporting a probable causal relationship between pomalidomide and the pulmonary event [20]. The score was driven by previous reports of similar pulmonary toxicity, temporal association after pomalidomide exposure, improvement after drug discontinuation and corticosteroid therapy, lack of a more likely alternative cause, and objective evidence including CT abnormalities and BAL eosinophilia (Table 2).

**Table 2:** Naranjo adverse drug reaction probability scale applied to pomalidomide

Naranjo item	Answer	Score
Previous conclusive reports on this reaction?	Yes	+ 1
Did the adverse event appear after the suspected drug was administered?	Yes	+ 2
Did the reaction improve after the drug was discontinued or a specific antagonist was given?	Yes	+ 1
Did the reaction reappear after readministration?	Not done	0
Are there alternative causes that could on their own have caused the reaction?	No likely alternative cause	+ 2
Did the reaction reappear after placebo?	Not applicable	0
Was the drug detected in blood or other fluids in toxic concentrations?	Not applicable	0
Was the reaction more severe when the dose was increased or less severe when the dose was decreased?	Unknown	0
Did the patient have a similar reaction to the same or similar drugs previously?	No/unknown	0
Was the adverse event confirmed by objective evidence?	Yes: CT abnormalities and BAL eosinophilia	+ 1
Total score	Probable adverse drug reaction	7

A comparison with previously reported cases of pomalidomide-related lung injury is provided in Table 3.

**Table 3:** Previously reported cases of pomalidomide-related eosinophilic pneumonia or related lung injury

Author, year	Time to onset	BAL findings	Outcome
Geyer et al. 2011, case 1 [12]	480 d	Normal	Rapid improvement; relapse after reintroduction
Geyer et al. 2011, case 2 [12]	120 d	45% eosinophils	Rapid improvement; reintroduction without recurrence
Sasieta Tello et al. 2013 [13]	7 d	Alveolar hemorrhage	Rapid improvement; relapse after reintroduction
Modi et al. 2015 [14]	8 mo	NR	Progressive improvement; relapse after reintroduction
Gajic et al. 2018 [15]	22 mo	30% lymphocytes	Progressive resolution; persistent fibrotic lesions
Icard et al. 2021 [16]	6 d	71% lymphocytes	Rapid resolution
Vivien et al. 2023 [17]	2 mo	25% eosinophils	Rapid resolution; no recurrence during follow-up
Carter et al. 2024 [18]	NR	25% eosinophils	Clinical improvement reported
Present case	4 wk	32% eosinophils, 48% lymphocytes	Rapid clinical improvement; near-complete radiological resolution at 1 mo; sustained functional recovery at 12 mo

Abbreviations: *BAL* Bronchoalveolar lavage, *NR* Not reported, *d* days, *wk *weeks, *mo* months

Prompt recognition of drug-induced pulmonary toxicity is crucial. Continuation of the offending agent in the context of ongoing injury may lead to persistent or irreversible pulmonary impairment, including fibrotic sequelae [15]. Withdrawal of pomalidomide and early corticosteroid therapy in our patient led to rapid clinical recovery, near-complete radiological resolution at one month, and near-baseline gas transfer at 12 months.

Notably, daratumumab was maintained after pomalidomide withdrawal without recurrence of pulmonary symptoms. Daratumumab-induced interstitial pneumonitis has been reported [21], but, to our knowledge, no published case of daratumumab-induced eosinophilic pneumonia has been identified. In the present case, the absence of recurrence despite continuation of daratumumab argues against daratumumab as the primary culprit.

## Conclusion

This case highlights a rare but important adverse event associated with pomalidomide use. Clinicians should maintain a high index of suspicion for drug-induced eosinophilic pneumonia in patients presenting with new respiratory symptoms during therapy. Early diagnosis, discontinuation of the causative agent, and corticosteroid therapy when clinically indicated are key to achieving favourable outcomes.

## Data Availability

No datasets were generated or analysed during the current study.

## Abbreviations

BAL: Bronchoalveolar lavage
CT: Computed tomography
DLCO: Diffusing capacity of the lungs for carbon monoxide
DPD: Daratumumab, pomalidomide, and dexamethasone
FEV1: Forced expiratory volume in one second
FVC: Forced vital capacity
KCO: Carbon monoxide transfer coefficient
PCR: Polymerase chain reaction
TLC: Total lung capacity
